# Diagnostic accuracy of an automated microscope solution (miLab™) in detecting malaria parasites in symptomatic patients at point-of-care in Sudan: a case–control study

**DOI:** 10.1186/s12936-024-05029-3

**Published:** 2024-06-28

**Authors:** Muzamil M. Abdel Hamid, Abdelrahim O. Mohamed, Fayad O. Mohammed, Arwa Elaagip, Sayed A. Mustafa, Tarig Elfaki, Waleed M. A. Jebreel, Musab M. Albsheer, Sabine Dittrich, Ewurama D. A. Owusu, Seda Yerlikaya

**Affiliations:** 1 Department of Parasitology and Medical Entomology, Institute of Endemic Diseases, University of Khartoum, Khartoum, Sudan; 2 FIND, Geneva, Switzerland; 3 Department of Medical Laboratory Sciences, School of Biomedical and Allied Health Sciences, College of Health Sciences, University of Ghana, Accra, Ghana; 4 Malaria Control Program, Federal Ministry of Health, Khartoum, Sudan; 5 Faculty of Medical Laboratory Sciences, Sinnar University, Sinnar, Sudan; 6 Department of Biochemistry, Faculty of Medicine, University of Khartoum, Khartoum, Sudan; 7 Department of Infectious Disease and Tropical Medicine, Heidelberg University Hospital, 69120 Heidelberg, Germany

**Keywords:** Automated microscope, Artificial intelligence, miLab™, Malaria, Sudan

## Abstract

**Background:**

Microscopic detection of malaria parasites is labour-intensive, time-consuming, and expertise-demanding. Moreover, the slide interpretation is highly dependent on the staining technique and the technician’s expertise. Therefore, there is a growing interest in next-generation, fully- or semi-integrated microscopes that can improve slide preparation and examination. This study aimed to evaluate the clinical performance of miLab™ (Noul Inc., Republic of Korea), a fully-integrated automated microscopy device for the detection of malaria parasites in symptomatic patients at point-of-care in Sudan.

**Methods:**

This was a prospective, case–control diagnostic accuracy study conducted in primary health care facilities in rural Khartoum, Sudan in 2020. According to the outcomes of routine on-site microscopy testing, 100 malaria-positive and 90 malaria-negative patients who presented at the health facility and were 5 years of age or older were enrolled consecutively. All consenting patients underwent miLab™ testing and received a negative or suspected result. For the primary analysis, the suspected results were regarded as positive (automated mode). For the secondary analysis, the operator reviewed the suspected results and categorized them as either negative or positive (corrected mode). Nested polymerase chain reaction (PCR) was used as the reference standard, and expert light microscopy as the comparator.

**Results:**

Out of the 190 patients, malaria diagnosis was confirmed by PCR in 112 and excluded in 78. The sensitivity of miLab™ was 91.1% (95% confidence interval [CI] 84.2–95.6%) and the specificity was 66.7% (95% Cl 55.1–67.7%) in the automated mode. The specificity increased to 96.2% (95% Cl 89.6–99.2%), with operator intervention in the corrected mode. Concordance of miLab with expert microscopy was substantial (kappa 0.65 [95% CI 0.54–0.76]) in the automated mode, but almost perfect (kappa 0.97 [95% CI 0.95–0.99]) in the corrected mode. A mean difference of 0.359 was found in the Bland–Altman analysis of the agreement between expert microscopy and miLab™ for quantifying parasite counts.

**Conclusion:**

When used in a clinical context, miLab™ demonstrated high sensitivity but low specificity. Expert intervention was shown to be required to improve the device’s specificity in its current version. miLab™ in the corrected mode performed similar to expert microscopy. Before clinical application, more refinement is needed to ensure full workflow automation and eliminate human intervention.

*Trial registration* ClinicalTrials.gov: NCT04558515

**Supplementary Information:**

The online version contains supplementary material available at 10.1186/s12936-024-05029-3.

## Background

Malaria remains a major health concern in the tropics, especially in sub-Saharan Africa, despite significant improvements in malaria control and management in recent decades [1]. In Sudan, malaria is a serious public health problem with loss of livelihood and economic impact [2, 3]. Successful management of malaria in patients requires correct and timely diagnosis by detecting the malaria parasites in the blood smear and administering an effective treatment. Light microscopy has been the standard of reference for malaria diagnosis since the introduction of Giemsa stain in 1904 [4]. Main reasons behind its longstanding reign are its low direct cost and ability to detect, quantify, and differentiate malaria parasites. However, this major malaria diagnostic tool also has its well-recognized limitations; it is labour-intensive, time-consuming, and expertise-demanding [5]. Moreover, competence level of operators plays a decisive role in slide interpretation [6]. Efforts to standardize the quality have intensified within the last decade resulting in considerable improvements in parasitological diagnosis of malaria by microscopy [7]; however, these efforts also proved to be expensive and difficult to sustain, especially in settings where the number of malaria cases is in decline [4]. Besides, the quality of equipment and infrastructure used and the staining technique preferred often impact the results greatly, even with highly competent microscopists especially in detecting low parasitaemia [8].

With the aim of advancing the conventional microscopy by addressing its limitations, multiple developers have come up with innovative diagnostic solutions which combine features like automated smearing, staining, image acquisition, and/or analysis by artificial intelligence (AI)-based algorithms for the identification of *Plasmodium* parasites [9–11]; however, very few of these solutions propose a fully-integrated, sample-to-result approach. One such example is the Micro-Intelligent Laboratory (Noul Inc, Ltd., Republic of Korea), referred to as miLab™, a technology platform that provides rapid (< 30 min), automated, and standardized diagnosis for all human-infecting species of malaria [12]. The portable and battery-driven instrument automatically performs (i) sample preparation: peripheral thin blood smear, fixation, and staining using proprietary stamping technology using a disposable cartridge [13], (ii) digital imaging with high resolution and speed (500 × lenses and CMOS sensor) scanning all red blood cells (RBC) in 400 fields; and (iii) embedded AI-based analysis for parasite detection and quantification performed on a server-free central processing unit.

The current study aimed to perform a prospective validation of diagnostic accuracy of miLab™ in detecting malaria parasites in primary health care facilities in Sudan, as part of the Innovation Platform project of FIND, the global alliance for diagnostics [14].

## Methods

### Study design

This was a prospective, case–control diagnostic accuracy study. Both cases and controls were sampled from a single source population, patients with symptoms suggestive of malaria seeking clinical care in health facilities. A total of 100 malaria-positive and 90 malaria-negative patients based on the results of routine microscopy testing at the health care facility were screened for eligibility and enrolled consecutively.

### Sample size

The sample size was calculated assuming miLab™ would have a sensitivity of 93.75% and a specificity of 95.65%, each with a 95% CI of ± 5%, based on preliminary data from the developer using PCR as the reference standard. Additionally, due to the higher sensitivity of molecular methods over microscopy, up to 30% of samples identified as negative by microscopy were expected to be false negatives when verified by PCR [15].

A total of 190 participants were enrolled in the study. It was estimated that 100 patients positive for malaria (cases) by routine microscopy would need to be recruited for the evaluation of miLab™ to obtain a reliable estimate of the expected sensitivity, with 95% power of obtaining a 95% confidence interval (CI) of ± 10% or less, while allowing for procedural errors in 10% of all cases. In addition, it was estimated that 90 patients negative for malaria (controls) by routine microscopy would need to be recruited for the evaluation of miLab™ to obtain a reliable estimate of the expected specificity with 95% power of obtaining a 95% CI of ± 10% or less, while allowing for procedural errors in 10% of all controls and a false-negativity rate of 30% among controls as determined by the reference standard. The formula used for sample size calculation can be found in [16].

### Study area

Study participants were enrolled between October 2020 and December 2020 at two primary health care centers at Gezira Slanj (GS) and Alsororab (SOR) in rural Omdurman, 40–50 km north of Khartoum (Suppl. Fig. S1). Both sites are endemic for *Plasmodium falciparum* and *Plasmodium vivax* malaria [17]. Malaria transmission is seasonal, occurring twice a year. The first season occurs during the short rainy period, which peaks from July to September. The plantation irrigation in the area causes the second season, which runs from October through March.

### Inclusion and exclusion criteria

Patients were included in the study if they were five years of age or older, had a malaria status (positive or negative) determined by routine microscopy at the health facility where they were presenting, freely agreed to participate by signing an informed consent form (adults aged 18 years and older and parent/legal guardian of a child) and providing assent (children aged 13–17 years), and were willing to provide a finger prick blood sample at enrollment. Severely ill patients as defined by WHO guidelines or patients who had received malaria treatment during the preceding four-week period were excluded from this study [18, 19].

### Specimen collection, handling, and storage

**Fig. 1:**
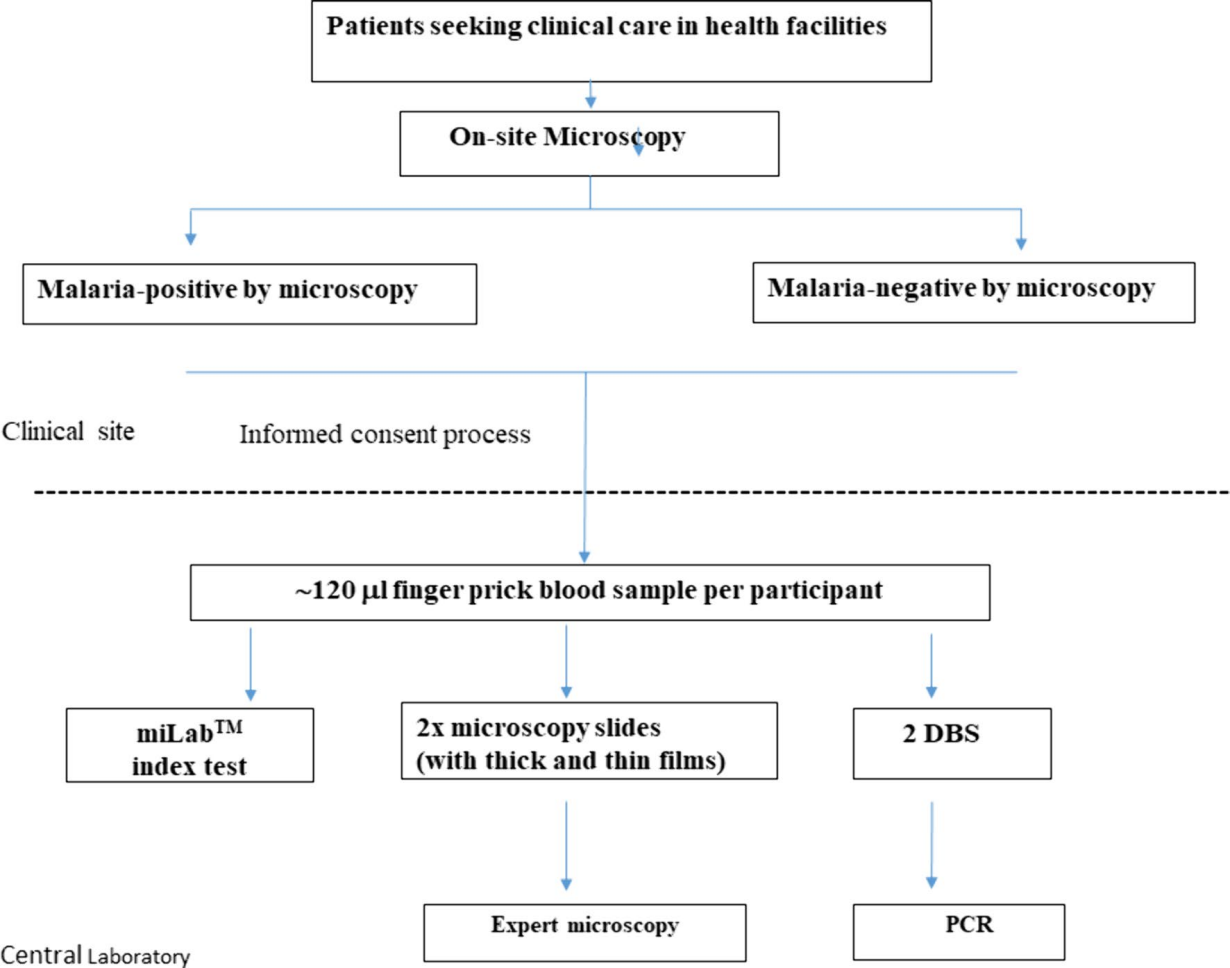
Flow chart of the study procedures

Capillary sampling was performed by trained laboratory personnel according to World Health Organization (WHO) guidelines on drawing blood [20]. A total of 120 µL fresh blood was collected from a finger prick: two microscopy slides with thin and thick blood smears were prepared using 15 µL, two dried blood spots (DBS) for DNA extraction were prepared using 100 µL, and 5 µL was used for miLab™ testing. Figure 1 describes the procedures that were performed during this study.

### Index test (miLab™)

Storage, handling, and testing by miLab™ were performed in accordance with the manufacturer’s instructions. Five microlitres of fresh capillary blood were directly loaded onto a single-use cartridge (Model: MDX1000 I P/N CMAA); Lot number 200827210226) which was then inserted into the cartridge stage of the miLab™ device (Serial number: N203 DMLA20010601; N205 DMLA19122001) (Suppl Fig. S2). A plastic glass slide was applied on the plastic cartridge before insertion. The device automatically performed thin blood smearing, staining, and image acquisition within minutes. The cartridge was automatically expelled after testing completion. The result screen displayed acquired images of parasitized (suspected) and non-parasitized (negative) RBC, along with the number of parasites and parasite count per microliter (µL) (Suppl Fig. S2). The miLab™ algorithm detected ring and trophozoite forms of all *Plasmodium* species but could not differentiate between them. Finally, the cartridge and other disposals were disposed of according to local regulations.

The operators received online training on how to operate miLab™ and were blinded to the comparator and reference standard results. In the corrected mode, the same operators reviewed the suspected results and categorized them as negative or positive. Supplementary Fig. S3 A, B & C shows RBC with or without parasites in images captured by miLab™. The parasite count per µL of blood was calculated using the following formula embedded in the device: Parasitaemia (parasite [p]/µL) = [(Ring # + Trophozoites # + Gametocytes #) / Total Number of RBC] × 5.00 × 1,000,000.

### Comparator test (microscopic examination)

As per standard protocols, thin and thick blood smears were stained with 3% freshly prepared Giemsa (RAL Diagnostics, France), and allowed to dry at room temperature for an hour [21, 22]. Thick films were used for detection of *Plasmodium* parasites, whereas thin blood films enabled identification of infecting species.

On-site study microscopy was conducted at the two health facilities (GS and SOR) by trained microscopists. Expert microscopy reading was performed at the central laboratory at the Institute of Endemic Diseases (IEND) by a WHO-certified expert (level I) microscopist. The parasite density was estimated by counting the number of parasites against 200 or 500 leucocytes depending on parasite density and assuming a density of 8,000 leucocytes per µL. Obare method calculator [23] was used to determine whether the parasite number calculated by the site microscopists and the expert microscopists were discordant. A third WHO-certified microscopist (level I) was included for reading the slides when there was discordance between the first and second microscopists.

### Reference standard (nested PCR)

DNA was extracted from a half piece of a DBS (25 µL) using QIAamp DNA extraction kit (Qiagen, Germany) following the manufacturer instructions. Nested PCR (PCR) for detection of *Plasmodium* parasite species was used as reference standard and performed at IEND following the protocols previously described [24]. Negative, no-template, and positive controls, which were kindly provided by the WHO malaria amplification test external quality assessment scheme (WHO-NAAT), were included in each assay. Nucleic acid extraction and subsequent PCR testing were carried out within three months of sample collection. Operators performing the reference test were blinded to the index test results.

### Baseline data and statistical analysis

Demographic and clinical data were recorded on a case report form by qualified medical doctors. A unique participant identifier was assigned to each study subject.

OpenClinica database was used for study trial data entry and monitored externally by FIND while SPSS 21.0 and MedCal softwares were used for statistical analysis. Sensitivity, specificity, and accuracy were calculated, together with 95% confidence intervals (CI), using Wilson’s score methods [25]. In order to compare miLab™ and expert microscopy against the reference standard, Cohen’s kappa (κ), a measure of concordance, was computed along with 95% CI. The concordance interpretation was as follows: κ ≤ 0 as no agreement, 0.01–0.20 as none to slight, 0.21–0.40 as fair, 0.41–0.60 as moderate, 0.61–0.80 as substantial, and 0.81–1.00 as almost perfect agreement. Bland–Altman analysis was used to assess the agreement between miLab™ and expert microscopy in quantifying parasite counts [26]. All values were expressed in the logarithmic form.

## Results

### General characteristics of study population

Table 1 provides a summary of the general characteristics of the study participants. All study participants, cases, and controls had median ages of 29 (range 5–75), 24 (range 5–75), and 33 (range 5–55) years, respectively. While more females were seen in the controls (N = 47; 52.2%), there were more males among the positive cases (N = 63; 63%). All participants presented with fever or a history of fever within the last 48 h. The geometric mean of parasite density among microscopy-positives as determined by expert microscopy was 17,657 parasites per µL of blood (p/µL) with a range from 351 to 192,560 p/µL (Table 1).

**Table 1:** Key characteristics of study population

	All	Malaria-positive	Malaria-negative
Number of participants*	190	100	90
Gezira Slanj	95	50	45
Alsororab	95	50	45
Median age (years) (median [range])	29 (5–75)	24 (5–75)	33 (5–55)
Female (number [%])	85 (44.7)	37 (37.0)	47 (52.2)
Temperature (°C) (mean [range])	37.3 (35.0–41.1)	37.1 (35.0–37.9)	37.5 (36.0–40.1)
Parasitaemia (geometric mean [range])	n/a	17,657 (351–192,560)	n/a
PCR	190	112	78
Pf	62	62	n/a
Pv	32	32	n/a
Pf/Pv	18	18	n/a
Expert microscopy	190	100	90
Pf	62	62	n/a
Pv	38	38	n/a
Pf/Pv	0	0	n/a
miLab™ (automated mode)	190	128	62
miLab™ corrected mode)	190	104	86

*Pf* *Plasmodium falciparum*, *Pv* *Plasmodium vivax*, *Pf/Pv* mixed infection, *n/a* not applicable

### Parasite identification by microscopy, PCR, and miLab™

Ninety (47.4%) samples were found to be negative for any *Plasmodium* infection, while 100 (52.6%) samples were found to be infected with *Plasmodium*, containing 62 *P. falciparum* and 38 *P. vivax*, according to routine microscopy confirmed by the expert microscopist (Table 1). Species identification by expert microscopy was identical to that of routine microscopy (Table 1).

The reference PCR method detected 112 positives and 78 negatives for malaria (Table 1, Additional file 1: Figure S2). Twelve of the microscopy-negatives were found to be positive by PCR, while all of the positives by microscopy were also positive by PCR (Table 1). Sixty-two (55.3%) *P. falciparum*, 32 (28.6%) *P. vivax*, and 18 (16.1%) mixed infections were detected among the PCR-positives (Table 1).

miLab™ identified 128 as suspected and 62 as negative in its automated mode, whereas 104 malaria-positives and 86 malaria-negatives were identified when corrected by the operator (Table 1).

### Diagnostic performance of miLab™ and expert microscopy in comparison to PCR

Table 2 displays the sensitivity, specificity, and accuracy of miLab™, routine microscopy, and expert microscopy compared to PCR. miLab™ demonstrated 91.1% (95% CI 84.19–95.64) sensitivity and 66.7% (95% CI 55.08–76.74) specificity when operating in automated mode. The specificity increased to 96.2% (95% CI 89.56–99.23%) in the corrected mode, but the sensitivity remained similar at 90.2% (95% CI 83.1–94.9). The accuracy of the device in the automated mode was 81.1% (95% CI 60.7–74.5) and 96.0% (95% CI 92.2–98.3) in the corrected mode.

**Table 2:** Diagnostic performance of miLab™ and expert microscopy in comparison to PCR

	N	TP	FP	FN	TN	Sensitivity % (95% CI)	Specificity % (95% CI)
miLab™ (automated mode)	190	102	26	10	52	91.1 (84.2–95.6)	66.7 (55.1–76.7)
miLab™ (corrected mode)	190	101	3	11	75	90.2 (83.1–94.9)	96.2 (89.6–99.2)
Expert microscopy	190	100	0	12	78	89.3 (82.0–94.3)	100 (95.4–100.0)
Routine microscopy	190	100	0	12	78	89.3 (82.0–94.3)	100 (95.4–100.0)

*N* total number, *TP* true positive, *FP* false positive, *FN* false negative, *TN* true negative, *CI* confidence interval

Sensitivity, specificity, and accuracy of expert microscopy in comparison to PCR were 89.3% (95% CI 82–94.3), 100% (95% CI 95.4–100), and 99.5% (95% CI 97.1–99.9), respectively (Table 2).

### Concordance of miLab™ with expert microscopy

The concordance between miLab™ and expert microscopy in the automated mode was substantial with a Cohen’s kappa of 0.65 (95% CI 0.54 to 0.76), while the concordance in the corrected mode was almost perfect with a kappa of 0.96 (95% CI 0.93 to 1.0).

### Inter-rater agreement of parasite counts between miLab™ and expert microscopy

**Fig. 2:**
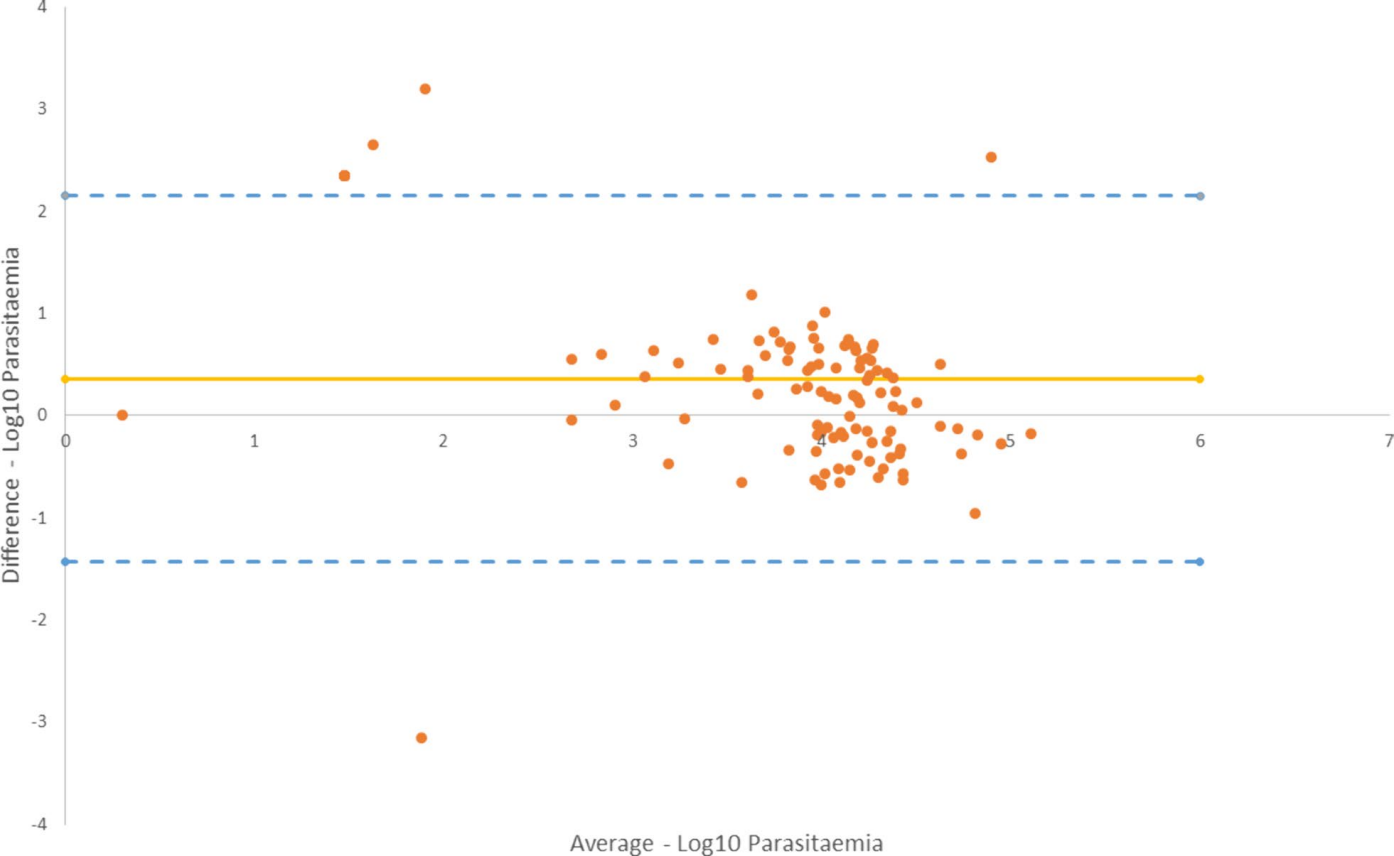
Bland–Altman plot for for parasite counts by miLab™ and expert microscopy (n = 112). The continuous line is theT log mean of differences of parasites counts while and dotted line is the limits of agreement with 95% confidence interval (dotted lines)

Bland–Altman analysis of the agreement between miLab™ and expert microscopy in quantifying parasite counts showed a mean difference of 0.359 with limits of agreement ranging from − 1.431 to 2.149 on a logarithmic scale (Fig. 2).

## Discussion

For proper treatment and efficient patient management, accurate diagnosis of malaria is crucial. Malaria diagnosis still relies heavily on microscopy in malaria-endemic settings; however, microscopy has significant drawbacks [27, 28], which are being attempted to be overcome by novel tools by introducing automation in the slide preparation, staining, digital image production and/or analysis [29, 30]. The current study aimed to assess the diagnostic accuracy of such a novel, fully-integrated, automated microscopy solution when used by intended end users at the point-of-care in a malaria-endemic setting. miLab™ demonstrated high sensitivity but low specificity at the manufacturer-specified threshold when compared to PCR. In its current fully-automated version, the concordance of miLab™ with expert microscopy was observed to be substantial. Overall, the parasite counts as determined by miLab™ and the expert microscopy differed by 2.3 fold.

Although numerous AI-based solutions for computer-aided reading of thick and/or thin films for the diagnosis of malaria have been proposed, these methods still call for manual slide preparation, and the high variability in slide quality in healthcare facilities is likely to have an impact on the final diagnosis' accuracy [31–33]. The use of datasets comprised of slides prepared in the field for the algorithm development may be able to address this [34–36]. Additionally, users of app-based solutions often have to manually move the objective in order to scan films and take pictures for analysis; as a result, the results are not significantly more rapid than those obtained through manual reading [37]. Alternatives exist, integrating automated reading, scanning, and analysis; however, manual slide preparation is still necessary. In a multi-center, diagnostic accuracy trial, one such example, the Motic EasyScan GO, demonstrated 91.1% sensitivity, and specificity 75.6% [11, 38]. When evaluated using a set of WHO malaria microscopy evaluation slides, the same system achieved WHO Competence levels 1 in detection accuracy, 2 in species identification, and 1 in quantification [11]. In its current version, miLab™ showed comparable sensitivity to EasyScan GO in our study, but lower specificity. It is likely that further algorithm training with additional datasets will help resolve miLab™'s current specificity problem. An opportunity for algorithm development exists with the corrected mode. In contrast to EasyScan GO, miLab™ integrates slide preparation into its system, ensuring that slide quality is maintained regardless of the setting or operator training level [11]. The performance of miLab™ in a multi-centre diagnostic accuracy trial as well as on the WHO evaluation slide set will need to be evaluated in the next step for a more accurate comparison, though. Moreover, the device’s usability in clinical settings remains to be investigated. However, it is worth noting that the device produces high quality images which can be used for educational and research purposes.

While it is promising that the device is digitized and open to AI learning and performance enhancement [11, 39], expert intervention remains necessary for acceptable performance in its current state. Nonetheless, with its shortened time to result (less than 30 min), the test still provides an alternative to conventional microscopic methods. Moreover, the parasite count generated by miLab™ did not correlate with that by expert microscopy, possibly due to differences in the mathematical models and the type of smears used for parasite quantification. The initial assumption made by microscopists about the number of white cells [40, 41] and the dependence of the entire process on relativity may also lead to limitations in the parasite count by manual microscopy. Conversely, the inclusion of gametocytes in miLab™ formula might lead to overestimation.

A considerable percentage of *P. falciparum* submicroscopic infection were observed, with *P. vivax* appearing as mixed infection in the PCR results. A previous study showed a high prevalence of *P. vivax,* reaching up to 26% in central and eastern Sudan [42]. Additionally, the study reported a high level of mixed infections detected by PCR, but not by microscopy, indicating a high prevalence of submicroscopic infection of both *P. falciparum* and *P. vivax* in the study area, consistent with the findings reported in this study.

There is one major limitation to consider when interpreting the results of this study. This was a prospective case–control study, so there could have been bias in the selection of participants. However, samples of cases and controls were taken from a single source population in order to minimize spectrum bias and limited-challenge bias. Moreover, operators performing microscopy and PCR were blinded to miLab™ results in order to avoid diagnostic review bias. To reduce classification bias resulting from incorrect identification of the infecting species and the relatively low sensitivity of other detection techniques like microscopy, PCR was selected as the reference standard. To prevent bias resulting from varying reference test methodologies used across sites, reference testing was conducted in a centralized reference laboratory. Additionally, to further prevent bias in clinical performance estimates due to partial verification; all study participants who underwent testing using miLab™ also underwent testing using the reference and comparator tests.

In conclusion, the miLab™ platform demonstrated potential for high sensitivity automated microscopy analysis for malaria diagnosis; however, its low specificity necessitates additional refinement prior to clinical application. To completely automate the workflow and eliminate human intervention, this will also be essential.

### Supplementary Information

**Additional file 1: Fig S1** Map showing the study area. **Fig S2** miLab™ platform and its display screen. **Fig S3** Images of parasitized and non-parasitized red blood cells produced by miLab™.

## Data Availability

All relevant data are within the manuscript. The datasets used and analysed during the current study are available from the corresponding author upon reasonable request.

## Abbreviations

AI: Artificial intelligence
CI: Confidence interval
DBS: Dried blood spot
DNA: Deoxyribonucleic acid
GS: Gezira Slanj
IEND: Institute of Endemic Diseases
miLab™: Micro-Intelligent Laboratory
NAAT: Nucleic acid amplification test
PCR: Polymerase chain reaction
RBC: Red blood cells
