# Supplementary figures and images for: Diagnostic accuracy of an automated microscope solution (miLab™) in detecting malaria parasites in symptomatic patients at point-of-care in Sudan: a case–control study
Source: Malar J. 2024 Jun 28;23:200. doi: 10.1186/s12936-024-05029-3 (PMC11212432; doi:10.1186/s12936-024-05029-3)

Fig. S1


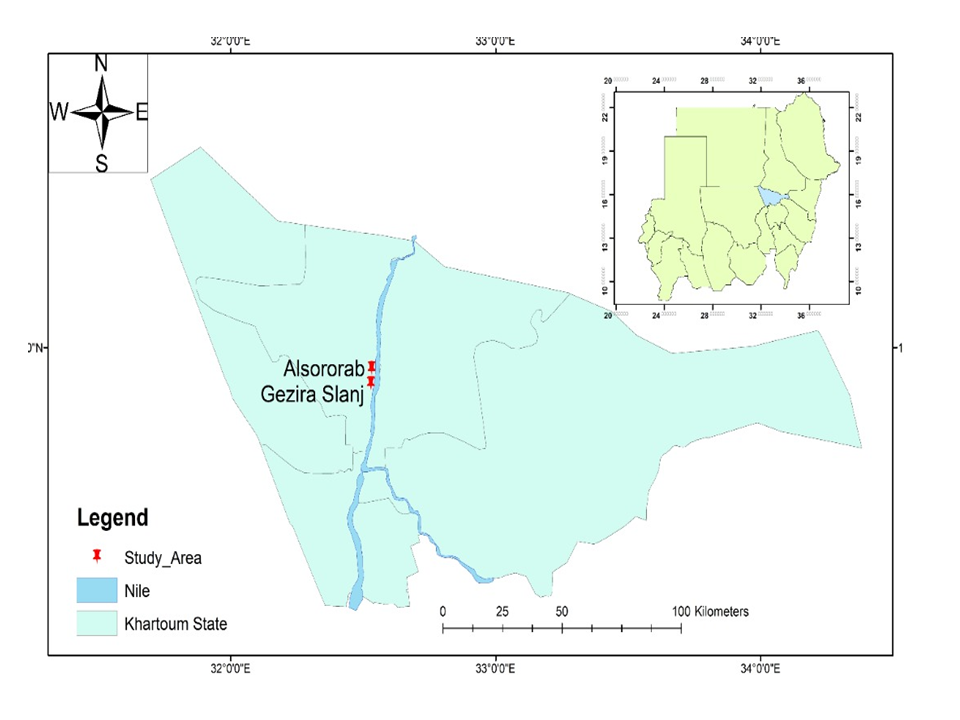


Fig. S2


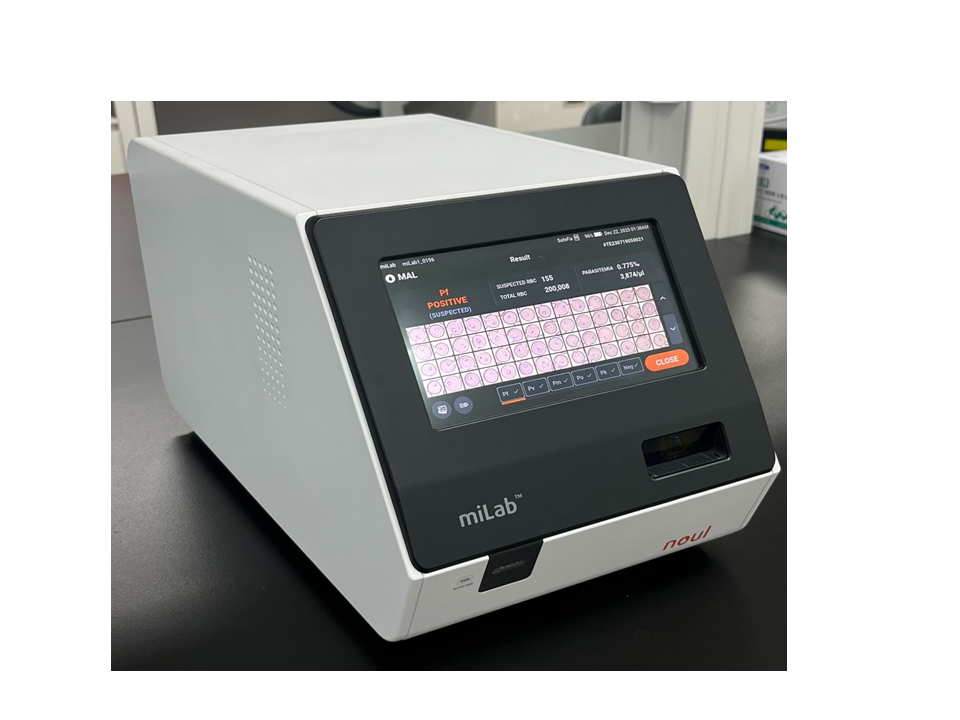


Fig. S3


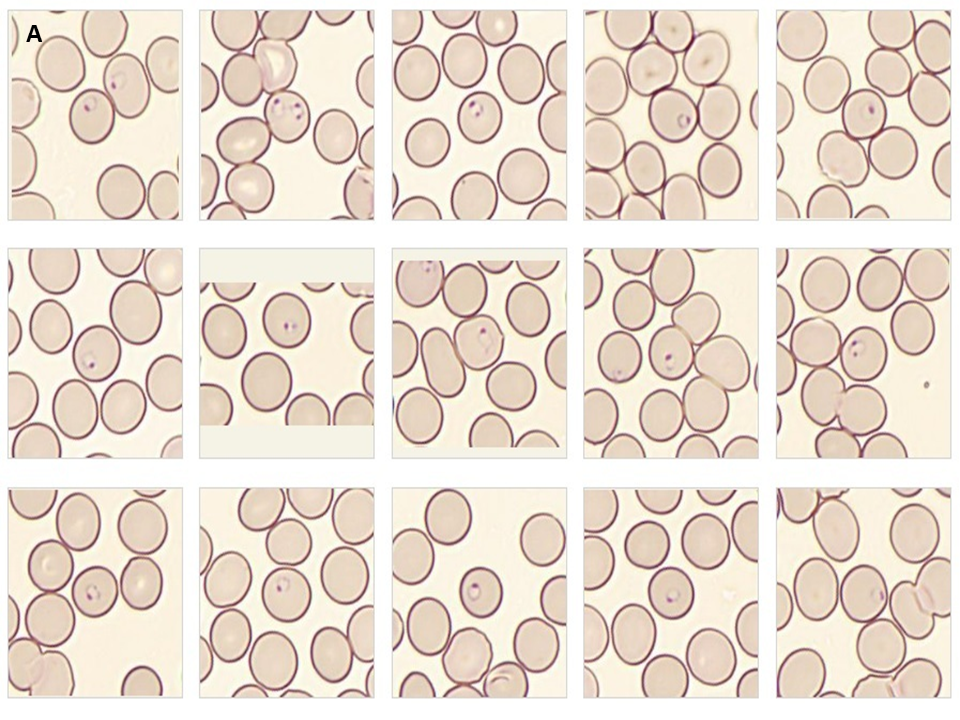


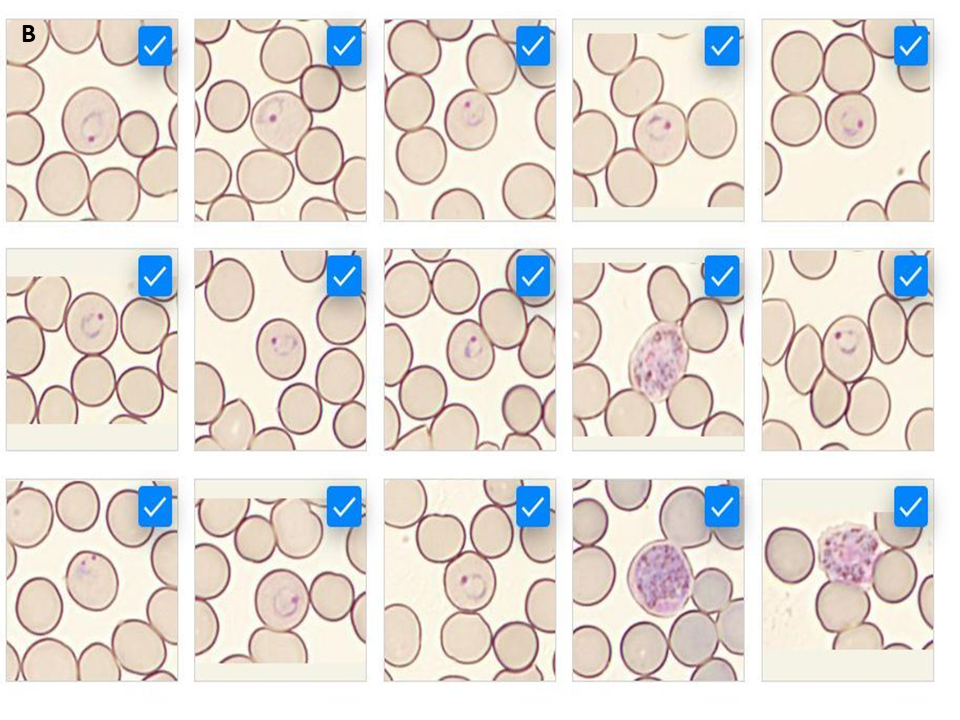


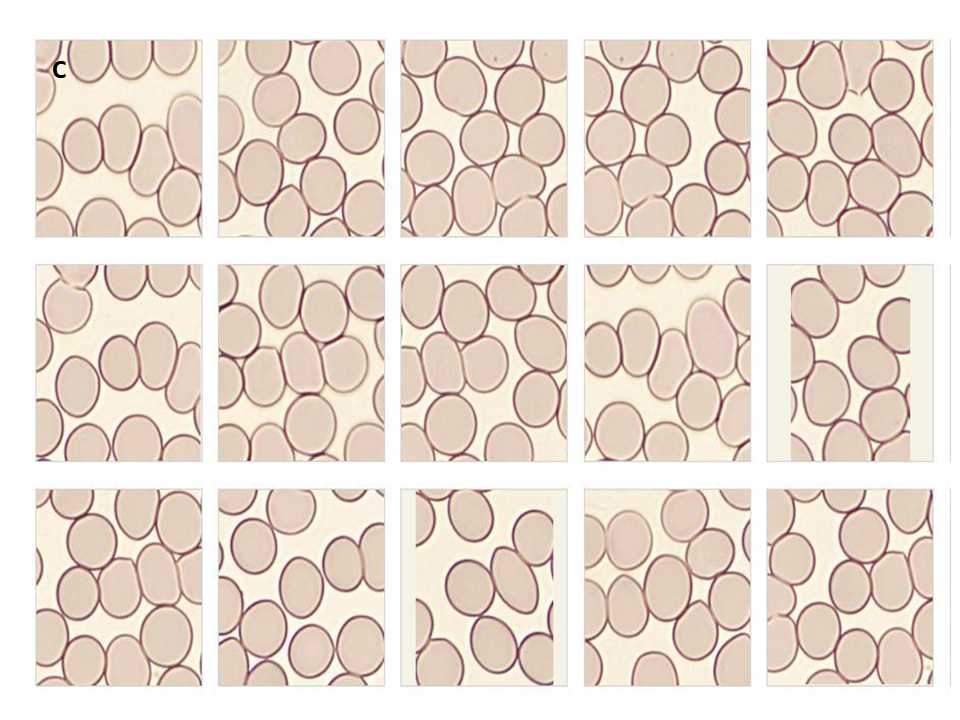

Supplement: Supplementary file 1 — Additional file 1: Fig S1 Map showing the study area. Fig S2 miLab™ platform and its display screen. Fig S3 Images of parasitized and non-parasitized red blood cells produced by miLab™. [file 12936_2024_5029_MOESM1_ESM.docx]
